# A dynamic study of VEGF-A siDOX-EVs trafficking through the *in-vitro* insert co-culture blood-brain barrier model by digital holographic microscopy

**DOI:** 10.3389/fonc.2024.1292083

**Published:** 2024-03-11

**Authors:** Parisa Shamshiripour, Mehrana Rahnama, Mehdi Nikoobakht, Fahimeh Hajiahmadi, Ali-reza Moradi, Davoud Ahmadvand

**Affiliations:** ^1^ Faculty of Medicine, Iran University of Medical Sciences (IUMS), Tehran, Iran; ^2^ Department of Molecular Imaging, Faculty of Advanced Technologies in Medicine, Iran University of Medical Sciences (IUMS), Tehran, Iran; ^3^ Department of Pathology, Shahid Beheshti Medical University (SBMU), Tehran, Iran; ^4^ Department of Biotechnology, Faculty of Biological Sciences, Alzahra University, Tehran, Iran; ^5^ Department of Neurosurgery, Iran University of Medical Sciences (IUMS), Tehran, Iran; ^6^ University of California San Francisco, Cellular Molecular Pharmacology School, School of Medicine, San Francisco, CA, United States; ^7^ Department of Physics, Institute for Advanced Studies in Basic Sciences, (IASBS), Zanjan, Iran; ^8^ School of NanoScience, Institute for Research in Fundamental Sciences (IPM), Tehran, Iran

**Keywords:** glioblastoma, blood-brain barrier, blood tumor barrier, drug delivery, co-culture

## Abstract

**Introduction:**

Modeling the blood-brain barrier has long been a challenge for pharmacological studies. Up to the present, numerous attempts have been devoted to recapitulating the endothelial barrier *in vitro* to assess drug delivery vehicles’ efficiency for brain disorders. In the current work, we presented a new approach for analyzing the morphometric parameters of the cells of an insert co-culture blood-brain barrier model using rat brain astrocytes, rat brain microvascular endothelial cells, and rat brain pericytes. This analytical approach could aid in getting further information on drug trafficking through the blood-brain barrier and its impact on the brain indirectly.

**Methods:**

In the current work, we cultured rat brain astrocytes, rat brain microvascular endothelial cells, and rat brain pericytes and then used an insert well to culture the cells in contact with each other to model the blood-brain barrier. Then, the morphometric parameters of the porous membrane of the insert well, as well as each cell type were imaged by digital holographic microscopy before and after cell seeding. At last, we performed folate conjugation on the surface of the EVs we have previously tested for glioma therapy in our previous work called VEGF-A siDOX-EVs and checked how the trafficking of EVs improves after folate conjugation as a clathrin-mediated delivery setup. the trafficking and passage of EVs were assessed by flow cytometry and morphometric analysis of the digital holographic microscopy holograms.

**Results:**

Our results indicated that EVs successfully entered through the proposed endothelial barrier assessed by flow cytometry analysis and furthermore, folate conjugation significantly improved EV passage through the blood-brain barrier. Moreover, our results indicated that the VEGF-A siDOX-EVs insert cytotoxic impact on the cells of the bottom of the culture plate.

**Conclusion:**

folate-conjugation on the surface of EVs improves their trafficking through the blood-brain barrier and by using digital holographic microscopy analysis, we could directly assess the morphometric changes of the blood-brain barrier cells for pharmacological purposes as an easy, label-free, and real-time analysis.

## 1 Introduction

The blood-brain barrier is a major challenge for brain drug delivery. To date, numerous mechanisms have been devoted to generating strategies to bypass the BBB or to increase its permeability to deliver drugs to the brain for neurological or neurosurgical disorders (1). using hyperosmotic mannitol, physical permeation by using ultrasound, and using vasoactive agents such as bradykinin are some of the examples of such efforts which in turn may increase the risk of co-morbidities such as infection. Using bypassing strategies such as the end-feet of the olfactory nerve or trigeminal nerves also called the intranasal route is another proposed strategy that has garnered much attention in recent years (2–4). However, this strategy also needs optimal nano-delivery system characteristics fine-tuned and based on the nerve fibers’ route and physiology, the delivered cargo may be released at different brain areas or may be distributed diffusely which is still a challenge. Hence, strategies focusing on the dynamics and molecular interactions of endothelial cells at BBB are still among the most famous and well-known methods to design engineered nano vehicles.

Extracellular vesicles (EVs) are small nano-sized vesicles shed in the biofluids that act as potent drug delivery systems for brain delivery (5–11). Previous literature notes that EVs released from mesenchymal cells (12–15) or immune cells (16–20) are actually miniature of their parental cells and could insert direct anti-/pro-inflammatory impacts on the recipient cells by delivering a vast variety of intrinsic cargos such as fragmented DNAs, RNAs, and small peptides which could in turn change or modulate the function and physiology of the recipient cells. Previous literature suggests that EVs pass through endothelial barriers such as BBB (21–25), and consequently, much research has been devoted to deciphering how exosomes actually pass through the BBB ultra-structurally (26, 27). In the current work, we evaluated the passage of EVs derived from dendritic cells loaded with therapeutic cargos for GBM and the dynamic morphometric changes of the BBB cells in an insert well *in-vitro* model of BBB and investigated how folate conjugation could enhance the trafficking of EVs across the BBB.

*In-vitro* BBB models provide fast and easy analysis of the drug delivery systems for the brain and have garnered much attention in recent years. The BBB models should be designed in such a way that they provide optimal resemblance to BBB physiology. Among the static models, the ones using insert porous membranes are well-known (28–30). A main concern for such models in the previous literature is that the proposed models do not fully reflect the cellular dynamics due to a lack of advanced imaging modalities to assess cell dynamic changes during treatment. Digital holographic microscopy (DHM) provides label-free, fast, quantitative, and real-time morphometric analysis of biological phenomena (31–33) such as cells seeded on transparent membranes to model BBB and hence, we hypothesized that by using this microscopy technique, we could more accurately decipher the dynamic changes of cells in the BBB model after treatment with our theranostic vehicle VEGF-A siDOX-EVs.

Furthermore, we hypothesized that by conjugating folate on EVs surface, we could enhance the uptake of EVs by brain endothelial cells. Mounting the previous evidence, the folate receptor is upregulated in brain microvasculature and consequently, folate could act as a potent targeting moiety for brain delivery of therapeutic vehicles across the BBB (34–36). Moreover, some precious pieces of previous evidence have also highlighted the upregulation of folate receptors in gliomas and its potency for the smart delivery of anti-cancer agents to cancerous brain cells (37). To this end, in the current work, we introduced a novel technique to assess the passage and trafficking of EVs across the insert BBB model comprising rat brain astrocytes, rat microvascular endothelial cells, and rat brain pericytes using DHM and also assessed how folate conjugation impacts the uptake of EVs in the BBB model. This study is a preliminary backbone for further analyses to recapitulate BBB dynamics after interacting with therapeutic nanovesicles for drug delivery purposes for a vast variety of brain disorders and could be further translated to pre-clinical settings for pharmacological purposes.

## 2 Materials and methods

### 2.1 RBMECs isolation and culture

RBMECs were isolated from the brain cortices of three Wistar rat neonates (about 15 g) using a combination of mechanical and chemical lysis. the rats were anesthetized with Ketamine/Xylazine (9:1) solution injection, decapitated, and their brains extracted. The brains were placed in a sterile petri dish with ice-cooled dissection buffer (PBS, 1 M, pH 7.4; 1% FBS; and 10% penicillin-streptomycin). Under a stereomicroscope, the meninges, olfactory bulb, cerebellum, and large vessels were removed and the brains were smashed to isolate the cortices. Then the cortices were lysed by scalpel and by adding 1 ml of Trypsin-EDTA for 10 min at 37°C in a 5% CO2 incubator. To inactivate the Trypsin-EDTA, 2 ml of DMEM-F12 (2-4% FBS; 1% penicillin-streptomycin) was added, and the solution was to homogenize it. the solution was transferred to a 15 ml sterile falcon tube and Ficoll gradient endothelial cell isolation (Ficoll to cell suspension ratio, 3:1) was performed. The tube was centrifuged at 1200 rpm for 5 min at room temperature and collected the EC-containing plaque. The plaque was washed three times with PBS (1 M, pH 7.4) by centrifuging at 1200 rpm for 5 min at room temperature. The pellet was resuspended in 1 ml of DMEM-F12 (2-4% FBS; 1% penicillin-streptomycin) and transferred to a T75 ploy-L-lysin-coated flask with 9 ml of DMEM-F12 (10% FBS; 1% penicillin-streptomycin). The isolated cells were cultured for a week until they reached about 75% confluency and were characterized by CD31 flow cytometry.

### 2.2 Isolation and culture of astrocyte cells

To culture astrocytes, a combination of cortical cells was isolated from the cortices of male Wistar rat pups aged between 1-10 days. Four rat pups were required to obtain enough astrocytes. The cortex tissue was dissected from the brain and mechanically dissociated with a sterile surgical blade. The dissociated cortex was then treated with 3 ml of 25% trypsin and incubated in a CO2 incubator for 15 minutes to digest the tissue. The trypsin was then neutralized with an astrocyte-specific culture medium. The cells were resuspended by pipetting and then centrifuged at 300 x g for 5 min in a centrifuge. The supernatant was removed by decanting, and the pellet was resuspended in 10 ml of astrocyte culture medium. The cells were further dissociated by pipetting vigorously with a 10 ml plastic pipette until no tissue pieces remained. The single-cell suspension was then transferred to DMEM containing 1% penicillin-streptomycin and 10% FBS. The cells were seeded in a 25 or 75-mm culture flask and incubated at 37°C with 5% CO2. The medium was changed every 3 days by replacing 2/3 of the volume with fresh medium. Once the cells reached confluence, they were purified by mechanical shaking. The flasks were placed in a shaker incubator and shaken at 180 rpm for 30 min to detach microglia. The supernatant containing microglia was discarded and replaced with fresh medium. The flasks were shaken again at 240 rpm for 6 hours to isolate oligodendrocyte precursor cells (OPCs). After 6 hours, only astrocytes and their precursors remained in the flasks. To ensure complete removal of OPCs, the flasks were shaken vigorously by hand for 1 min. The cells were detached from the flasks and washed with 5 ml of PBS. The cells were then treated with 3 ml of trypsin and inactivated with 6 ml of astrocyte medium. The cell suspension was transferred to a Falcon tube and centrifuged at 180 x g for 5 min. The cells were plated in T75 culture flasks and incubated at 37°C in the CO2 incubator. On day 14 after the first split, the FBS concentration was reduced from 20% to 10%. One T75 flask yielded approximately 1.5-2 x 106 cells after the second split.

### 2.3 Isolation and culture of pericyte cells

Isolation and culture of RBPs were performed by subculturing of primary endothelial cells isolated by the protocol by M Heyba et al. (38). Afterward, cells were immunostained for PDGFRb as a positive pericytic marker.

### 2.4 Immunocytochemistry of GFAP, PDGFRb, and PECAM1

A total of 2*10^5^ RBMECs, RBAs, and RBPs were grown in complete condition media within 24-well plates, following the protocol outlined previously. The cells were then fixed with 4% formaldehyde for 30 minutes before being rinsed twice with PBS. To permeabilize the cells, a 0.3% Triton X-100 solution in PBS was applied for 30 minutes, followed by blocking with 10% goat serum in PBS. The cells were then immunostained overnight at 4 °C using primary antibodies against PECAM1 (CD31), GFAP, and PDGFRb diluted as recommended by the manufacturer. FITC-conjugated secondary antibodies were subsequently added at the appropriate dilution in PBS, and incubated in darkness for 60 minutes at 37 °C. After washing with PBS, the cells were mounted with DAPI, and fluorescent microscopy was performed.

### 2.5 Setting up the co-culture system

To create the BBB model, we started by applying a poly-L-lysine solution to the underhang. Next, we added 30,000 pericytes to the underside of the hang using a small amount of complete culture media (about drops of DMEM F12, supplemented with 10% FBS, 1% penicillin-streptomycin). Once the pericytes were attached to the coated porous insert culture hang (after one day), we tested two cellular densities for RBMECs; 30,000 RBMECs or 15000 on the upper hang. The RBMECs shared the same media with pericytes (1 ml of complete medium; DMEM F12, supplemented with 10% FBS, 1% penicillin-streptomycin) for one day. Finally, we added 30,000 RBAs to the wells to complete the BBB model. All the cultured cells shared the same medium at last.

### 2.6 Digital holographic microscopy image acquisition

To perform digital holographic microscopy (DHM) of transparent samples, we used a Mach-Zehnder interferometer. The interferometer divided a single light source into two parallel beams, which were then combined to produce interference patterns. To enable off-axis holography, we introduced a small angle between the two beams.

We aligned the optical paths of the two beams in the interferometer by placing a compensating cell made of the same glass material as the test cell in the reference beam. A 5 mW MEOS laser beam (632.8 nm) was expanded by a beam expander (BE) and split into two equal beams by a 50:50 beam splitter (BS1). One beam was reflected by BS1 and directed toward the sample (S) by a mirror (M1) and a condenser (C). The sample transmitted the beam with its information encoded. A 20X Olympus microscope objective (MO1, NA = 0.65, WD = 0.17 mm) collected the transmitted beam and passed it through BS2 to a digital camera (from Thorlabs, DCC1545M, 8-bit dynamic range, 5.2 μm pixel pitch).

The other beam transmitted by BS1 served as the reference beam and was reflected by a mirror (M2) and BS2 to interfere with the object beam on the camera. An off-axis DHM setup was achieved by introducing a slight angle between the reference and object beams. The interference patterns recorded by the camera were known as holograms and were numerically reconstructed using the angular spectrum propagation method in scalar diffraction theory. A second Olympus microscope objective (MO2), identical to MO1, was used to adjust the curvature of the interfering beams. A neutral density filter (NDF) was used to match the intensities of the two beams for optimal fringe contrast.

The sample was placed on a microscopy stage for imaging. The recorded holograms were numerically reconstructed to obtain the sample information.

### 2.7 Folate conjugation into EVs surface and EVs recharacterization

In this work, we used the EVs isolated in our previous work, loaded with VEGF-A siRNA and Doxorubicin named VEGF-A siDOX-EVs as a theranostic agent for glioma therapy to check the dynamic interaction of that treatment with BBB cells in an *in-vitro* insert well model. In order to conjugate folate (FA) to DC-EVs surface, the surface of exosomes was modified by amin-reactive NHS-ester-activated FA. In brief, FA (0.2g, 0.45 mmol) and DCC (0.18 g, 0.9 mmol) were mixed in 8 ml of DMSO in the presence of triethylamine (50.6 mg, 0.5 mmol)and were kept for 1 hour in a gentle shaker in dark at RT. Afterward, NHS (0.11g, 0.9 mmol) was added and the mixture was stirred overnight in dark at RT and was filtered to exclude the insoluble byproduct, decyclohexyl urea, and the resulting filtrate was precipitated using diethyl ether, and the crude product was washed with dihydrofuran to obtain the NHS-ester activated FA which was then dissolved in 1% DMSO-containing PBS (1mg/ml) and was mixed with 2 mg of exosomes at RT and the mixture was stirred overnight to obtain FA-exosomes. then, the mixture was subjected to a three-times wash with PBS and buffer exchange, and afterwards, recharacterization was performed. Afterward, EVs were again characterized for size and morphology by DLS and SEM to detect any possible aggregations after conjugation.

## 3 Results

This section is devoted to a detailed description of the results obtained from DHM analysis of the proposed blood-brain barrier. At first, we prepared the cells we needed for modeling the neurovascular unit (astrocytes, endothelial cells, and pericytes). Then, we set up the co-culture by using an insert well, and afterward, we proceeded to prepare the DHM setup and conjugate the exosomes with folate. In the next step, we assessed the cellular uptake of exosomes (folate conjugated or unconjugated) loaded with doxorubicin or FAM-labeled siRNA by flow cytometry and then visualized the barrier-exosome interactions by DHM. At last, we performed quantitative analyses from DHM results to compare the impact of each treatment on BBB functionality and cellular viability. We report our results in four subsections as follows:

(1) BBB Model Generation.
(2) DHM Setup, Image Acquisition, and Reconstruction of the BBB Model.
(3) Folate Conjugation, Recharacterization of EVs, and Visualization of EVs by DHM.
(4) Assessment of EV uptake and their impact on BBB cellular functions.

### 3.1 BBB model generation

#### 3.1.1 Rat brain astrocyte culture and characterization by GFAP ICC

**Figure 1:**
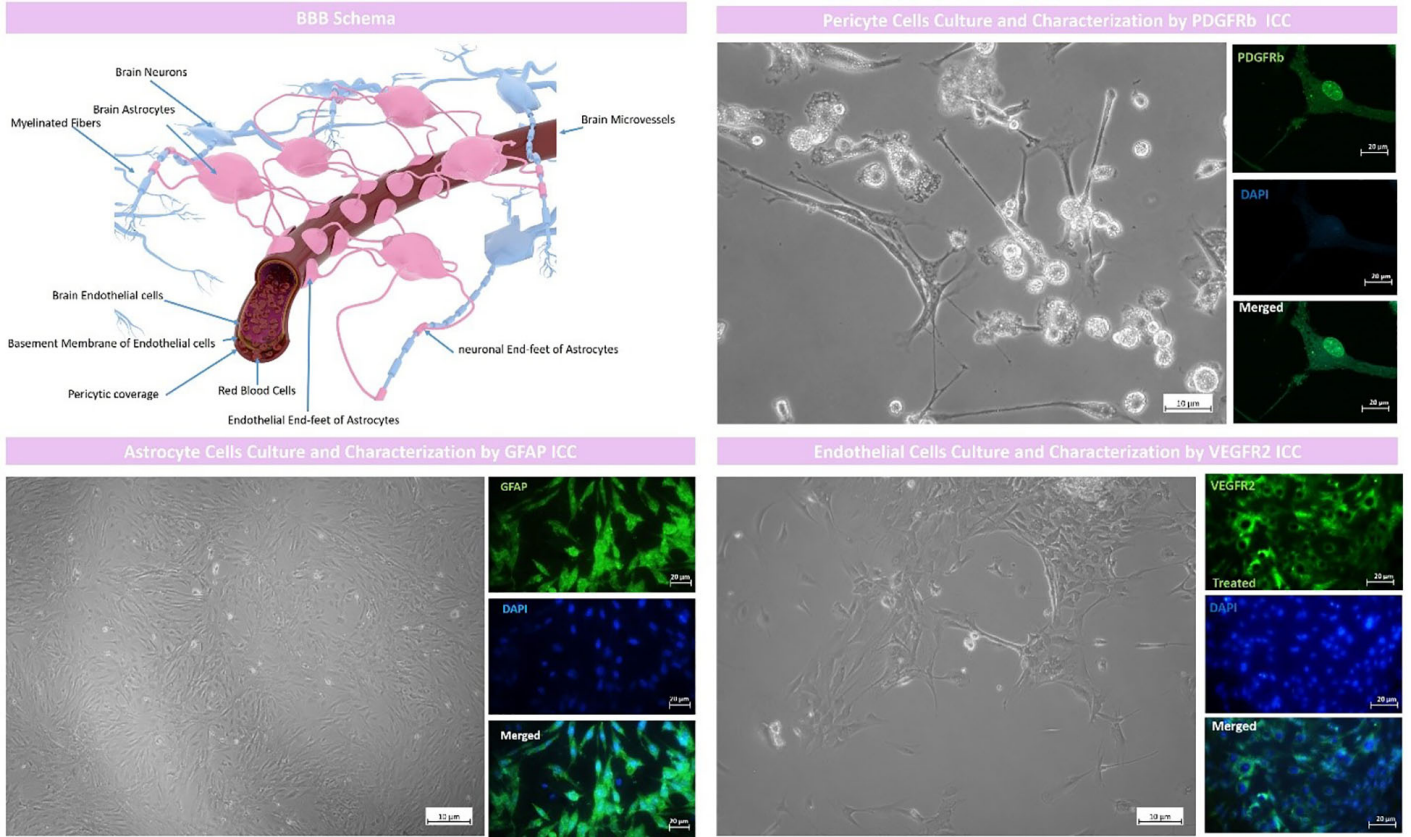
BBB model schema and cell culture and characterization. The proposed BBB model comprised of rat brain astrocytes (RBAs), rat brain microvascular endothelial cells (RBMECs) and rat brain pericyte cells (RBPs). RBAs were characterized by GFAP ICC with an expression of 93.25∓3.58, RBMECs were characterized by VEGFR2 ICC with an expression of 90.96∓6.51 and pericyte cells were characterized by PDGFR b ICC with an expression of 90.82∓3.85.

Rat brain astrocytes (RBAs) were successfully isolated by the protocol described in the methods section and represented a high expression of GFAP protein as a positive marker for astrocytes assessed by ICC of about 93.25∓3.58 suggesting that the isolated cells are purely astrocytes (**Figure 1**).

#### 3.1.2 Rat brain microvascular endothelial cells culture and characterization by VEGFR2 ICC

Brain endothelial cells were also isolated by the protocol we fully described in the methods section and represented high expression of endothelial cell marker VEGFR2 with a mean expression of 90.96∓6.51 which suggested that the isolated cells were purely of endothelial origin (**Figure 1**).

#### 3.1.3 Rat brain pericyte cells culture and characterization by PDGFRb ICC

As described in the methods section, we successfully cultured art brain pericyte (RBPs) cells and characterized them by PDGFRb ICC which represented a high mean expression of about 90.82∓3.85 which suggested that the isolated cells were purely pericytes (**Figure 1**).

#### 3.1.4 Setting up the co-culture system

**Figure 2:**
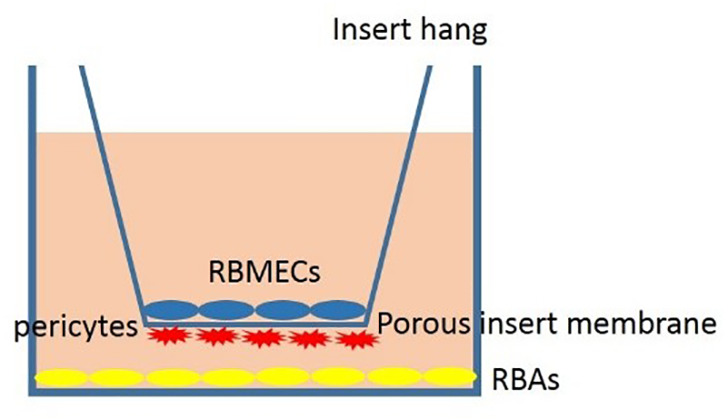
Schema of the proposed BBB model comprising rat brain astrocytes (RBAs), rat brain microvascular endothelial cells (RBMECs), and also rat brain pericyte cells (RBPs).

**Figure 3:**
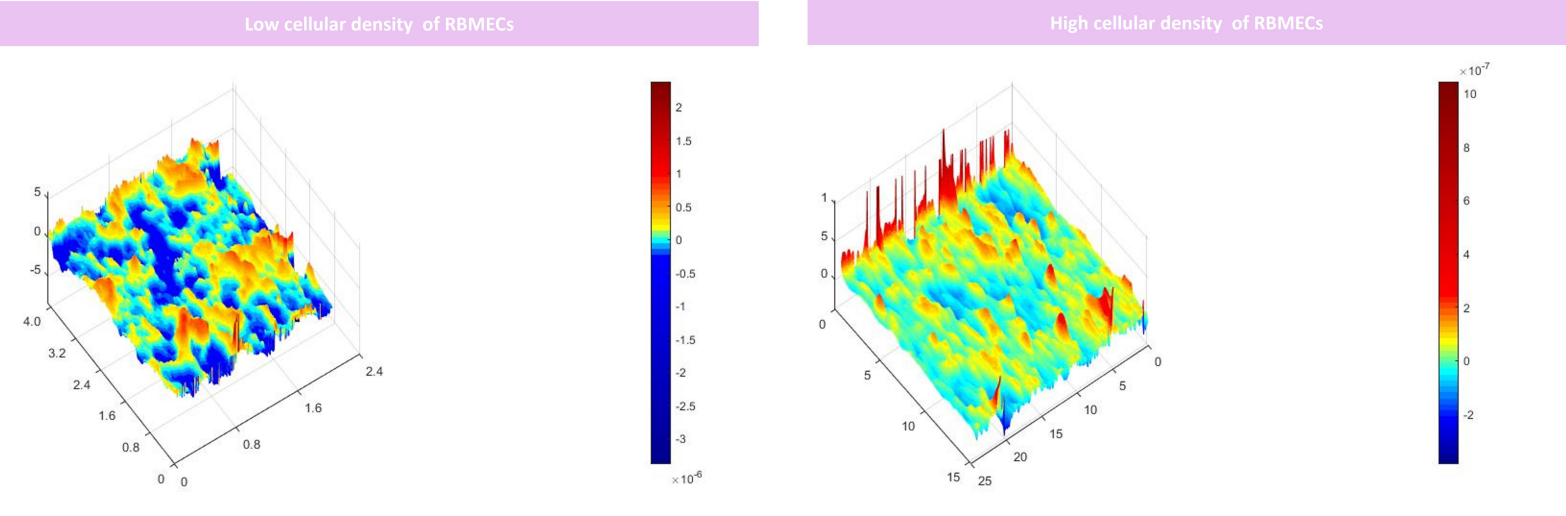
DHM analysis of the RBMECs different densities seeded on the porous membrane. Our results suggested that seeding 15000 cells did not provide adequate coverage for modeling BBB however by seeding 30000 cells optimized coverage was obtained. x, y, and z axis are in µm scale.

To model the BBB, firstly, the underhang was coated with poly-L lysine solution, and afterward, pericytes were seeded in a density of 15000 or 30,000 cells on the underside of the hang with a minimal amount of complete culture media. After the pericytes were attached to the coated porous insert culture hang (1 day), 30,000 RBMECs were seeded on the upper hang sharing the same media with pericytes (1 ml of complete medium; DMEM F12, supplemented with 10% FBS, 1% penicillin-streptomycin) for one day. Afterward, 30,000 RBAs were seeded on the wells to model BBB (**Figure 2**). Our findings demonstrate that the use of 15000 cells for BBB modeling provides insufficient coverage, while the use of 30000 cells results in optimal coverage. These results indicate that the effectiveness of BBB modeling is dependent upon the number of cells seeded, and that a higher number of cells results in improved coverage (**Figure 3**). Proper BBB modeling is critical for understanding the mechanisms of neurological diseases and for developing new treatments. Therefore, the use of an optimal cell number for seeding is essential for accurate and effective research.

### 3.2 DHM setup, image acquisition, and reconstruction of the BBB model

**Figure 4:**
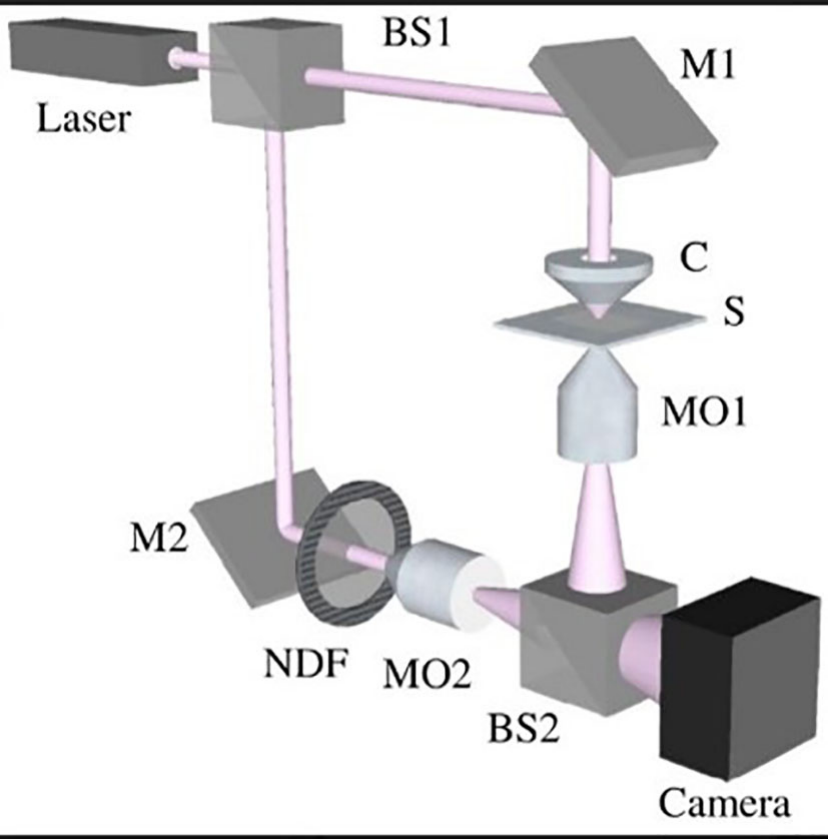
Schema of the digital holographic microscopy (DHM) setup based on a Mach–Zehnder interferometer arrangement.

**Figure 5:**
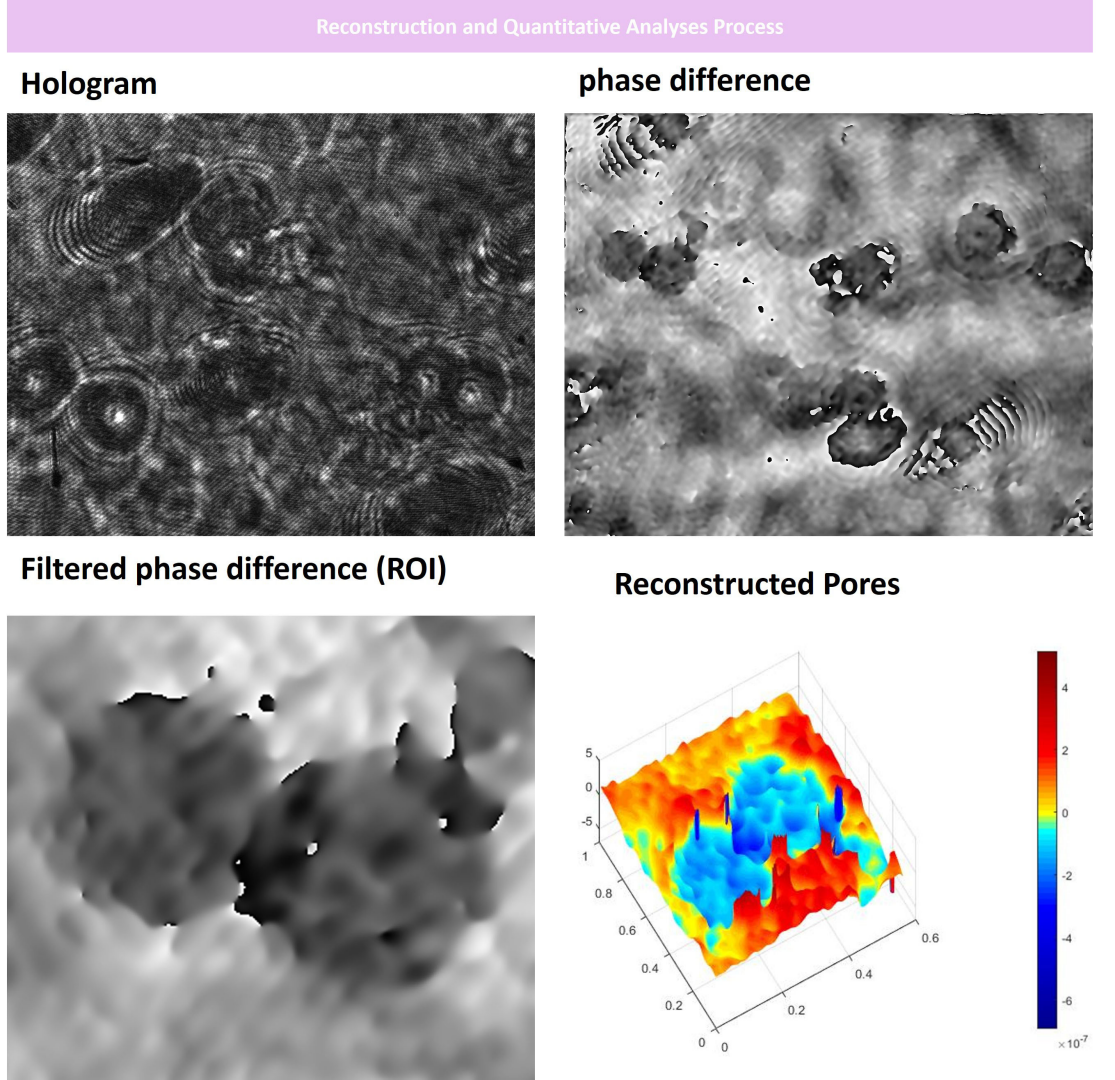
Digital Holographic Microscopy Analysis of the BBB insert well porous membrane before cells were seeded on. At first, we acquired hologram images and reference holograms. Then, we performed Fourier transformation and phase differentiation in MATLAB. Afterward, we filtered the differentiated images and finalized the 3D reconstruction process as depicted. Our DHM analyses indicated that the porous membrane used had an optimal pore size of about <0.4 mm for cells to adhere and communicate to each other forming the neurovascular unit. x, y, and z axis are in µm scale.

digital holographic microscopy (DHM) setup based on a Mach–Zehnder interferometer arrangement was set (**Figure 4**). Holograms were acquired by Thorlab imaging software with *20 magnification. Afterward, the gry format of the holograms was used for reconstruction. Initially, the phase images of each hologram were acquired by Matlab after numerical focusing. Then, we performed interferometry by choosing the optimal reference image for each reconstruction to obtain phase diff images. At last, we used a *3 filter and unwrapped the filtered phase diff to reconstruct the 3D format. As depicted in **Figure 5**, we successfully imaged RBAs, RBMEC, and the 3D morphology of the porous membrane before setting up the co-culture (**Figure 5**) and also after co-culturing to recapitulate the BBB structure.

### 3.3 Folate conjugation, recharacterization of EVs, and visualization of EVs by DHM

**Figure 6:**
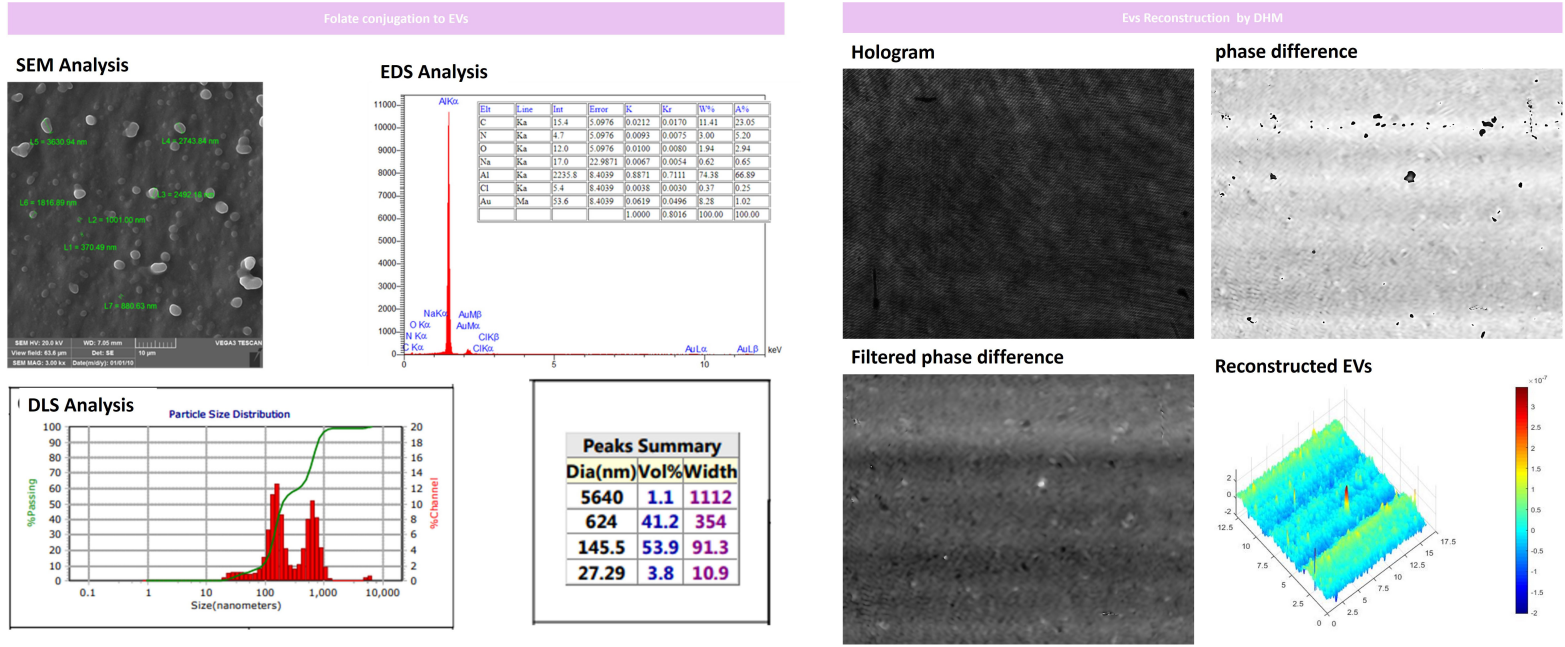
Folate-conjugation process and EVs recharacterization for size and morphology by DLS and SEM after folate conjugation. Our results indicated that EVs had intact size and morphology with minimal aggregation assessed and only 1% of the analyzed vesicles represented aggregation after conjugating folate on EV surfaces. Furthermore, we performed DHM analysis of EVs by fixing them on glass slides that represented round and spherical morphology. x, y, and z axis are in µm scale.

Our results indicated that the folate-conjugation process was performed successfully with optimal size and spherical morphology of EVs still intact. Only 1% of the analyzed vesicles represented aggregation as depicted in **Figure 6** assessed by DLS analysis after conjugation of folate on the EV surface. Moreover, we EVs 3D morphology was again checked by fixing them on glass slides for DHM analysis which represented round and spherical morphology (**Figure 6**).

### 3.4 Assessment of EV uptake and their impact on BBB cellular functions

#### 3.4.1 Uptake assessment by flow cytometry after folate-conjugation

Herein, we performed flow cytometry to assess how folate conjugation improves the uptake of EVs.

We needed to co-label the exosomes by PKH or DiI. However, we faced some issues due to the signal overlap of commercialized dyes such as DiI and Doxorubicin which both emitted in the red spectrum or PKH67 and FAM-labeled siRNA both emitting in the green spectrum for flow cytometry analysis. Moreover, previous literature suggested that EVs labeling with dyes such as PKH67 may be misinterpreted due to the micelle formation of hydrophobic PKH and hence, we decided to compare “Doxorubicin-loaded EVs” with “folate-conjugated Doxorubicin loaded EVs” and “siRNA-loaded EVs” with “folate-conjugated siRNA loaded EVs” to avoid labeling exosomes with hydrophobic dyes (e.g., PKH and DiI) to quantify the uptake rate (We used the fluorescent signal of Doxorubicin and FAM-labeled siRNA for quantification).

**Figure 7:**
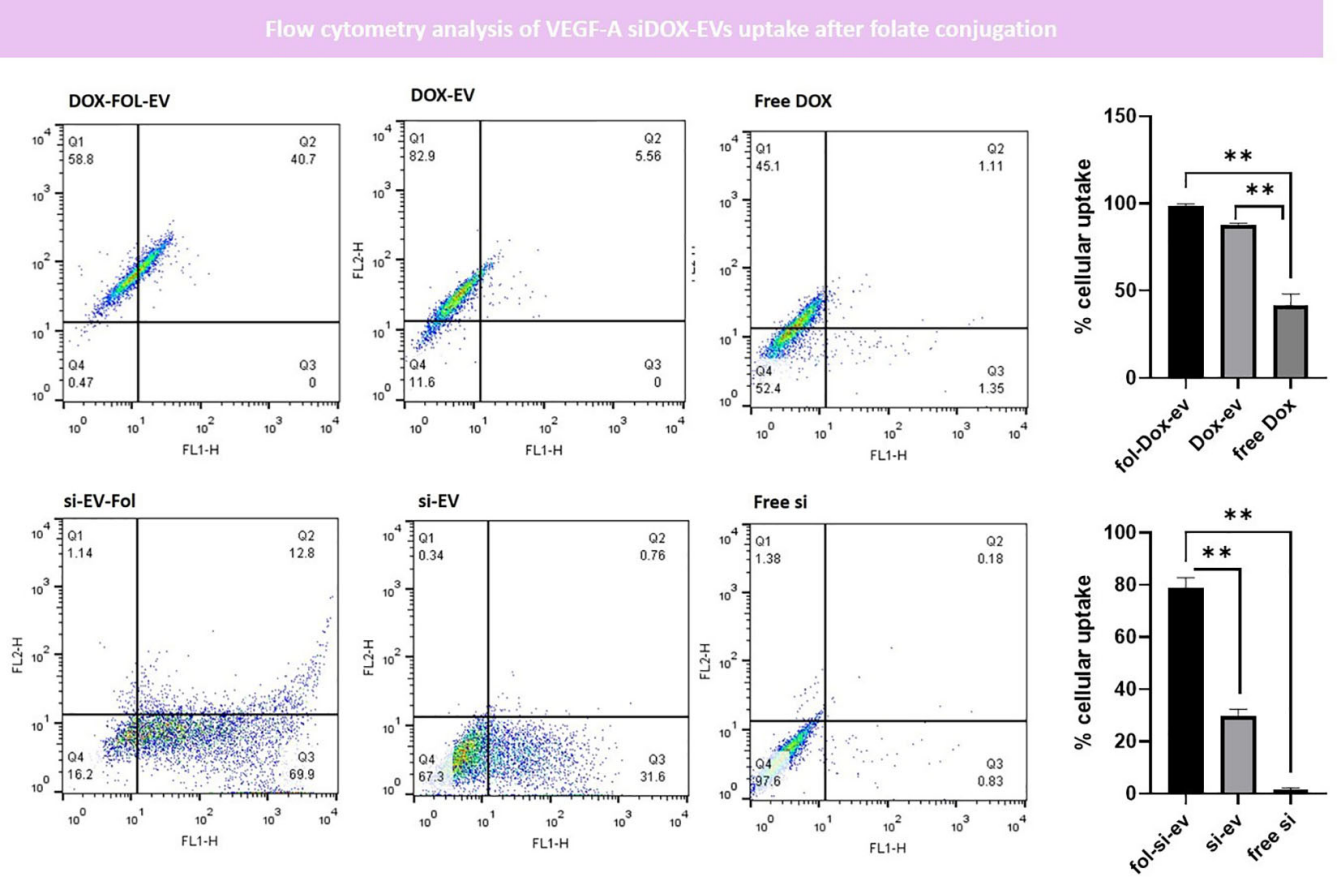
Flow cytometry analysis of EVs uptake after folate conjugation. Our data indicated that the uptake rate of fol-siEVs and fol-Dox-EVs are significantly higher than unconjugated ones (** = 0.001).

Our results indicated that on average, 78.94%∓5.31% of endothelial cells have uptaken folate-conjugated siRNA-Loaded EVs (fol-si-EVs) which was significantly higher than the uptake rate for unconjugated siRNA-loaded EVs (si-EVs) which was about 29.59%∓3.917% and also higher than the free siRNA treatment group (1.55%∓0.76%) significantly (p=0.01 and 0.02; respectively). This suggests that folate conjugation has significantly enhanced the uptake of EVs by endothelial cells which could act as a potent drug delivery system moving oligonucleotide drugs through the BBB. Comparable results were also obtained in Dox-loaded EVs. 98.81%∓0.97% of endothelial cells have uptaken folate-conjugated DOX-Loaded EVs (fol-DOX-EVs) which was significantly higher than the uptake rate for unconjugated DOX-loaded EVs (DOX-EVs) which was about 87.79%∓0.94% and also higher than the free siRNA treatment group (41.66%∓6.42%) significantly (p=0.007 and 0.05; respectively; **Figure 7**).

#### 3.4.2 Morphometric analysis of RBAs

**Figure 8:**
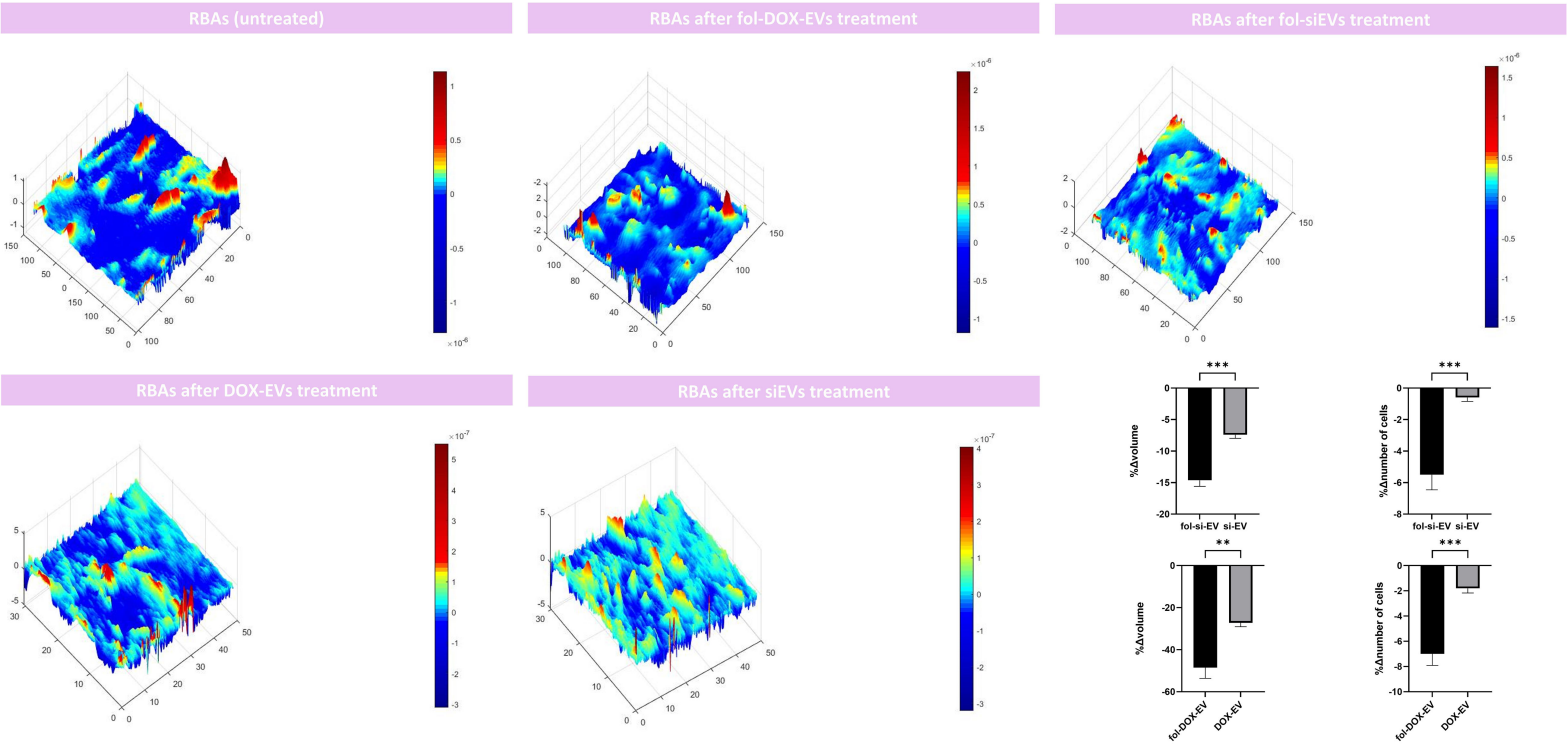
Morphometric analyses of the RBAs on the bottom of the co-culture model after treatment with si-EVs, Dox-EVs, fol-siEVs and fol-Dox-EVs. x, y, and z axis are in µm scale. (** = 0.001; *** = 0.0001).

After setting the BBB *in-vitro* culture model, we treated the insert wells with si-EVs, Fol-si-EVs, DOX-EVs, and fol-DOX-EVs to assess the impact of each of the formulations noted on the morphometry and number of cells to estimate the trafficking and efficiency of folate-conjugated EVs as drug delivery vehicles through the BBB. Our results indicated that the volume of RBA cells also decreased in the insert model up to 14.62%∓ 2.16%, 48.54%∓11.40% after Fol-si-EV and fol-DOX-EVs treatment; respectively. Moreover, the volume of RBA cells also decreased up to 7.406%∓ 1.26%, 27.25%∓4.026% after si-EV and DOX-EVs treatment; respectively. This suggests that the designed vehicle delivers the cargo to the adjacent cells after passing through the endothelial barrier and can induce necroptotic changes such as shrinkage at the cells seeded on the bottom of the culture plate. The number of RBAs also decreased after Fol-si-EV treatment and Fol-DOX-EVs by 4.4∓1.91 and -7∓1.82; respectively. Furthermore, 0.6%∓0.57% and 1.8∓0.836% decrease in the number of RBAs was also noted after si-EV and DOX-EVs treatment and cellular debris and fragmented cellular compartments appeared in DHM analyses. This piece of evidence also gives further credence to the hypothesis that our drug delivery system successfully passes through the endothelial barrier and inserts cytotoxic impact on adjacent cells in the bottom of the insert well (**Figure 8**).

## 4 Discussion

In the current work, we used EVs as delivery vehicles for BBB targeting and performed EV conjugation with folate to improve the BBB trafficking of our previously proposed theranostic vesicle “VEGF-A siDOX-EVs”. Mounting the previous studies, DC-EVs possess unique protein cargo which could insert significant impacts on combating glioma growth by activating the anti-tumor immune responses (20). Moreover, by using the exosomes derived from mesenchymal stem cells (MSC-EVs), potent regeneration was reported (39). Previous evidence suggests that cancers are wounds that do not heal (40) which also highlights the potential benefits of MSC-EVs for cancer therapy (41). As suggested by the International Society for Extracellular Vesicles (ISEV), exosomes are next-generation delivery systems in the future oncological practices however obstacles to their purity, high yield production, and safety have introduced some challenges in the way of their clinical translation currently (42, 43). These findings highlight the priority of exosomes to synthetic nanoparticles such as liposomes. Our results indicated that folate conjugation significantly enhances the cellular uptake of EVs by brain endothelial cells. This finding is in line with previous literature suggesting that folate conjugation improves the cellular trafficking of internalized cargo e.g. siRNAs by avoiding endosomal trapping (44) and could improve tumor targetability and uptake (45–48). In the current work, we proved that the therapeutic cargo reaches the RBA cells at the bottom of the culture plate after passing through the RBMECs, RBPs, and also the porous membrane and could insert cytotoxic impact on the cells. This is an interesting finding that gives further credence to the hypothesis that EVs could act as potent trojan horses delivering cargos of interest to the brain after passing through the BBB. RBA Cells show reduced volume and number which is suggestive of the necro-apoptosis process and eventually shrink. This morphometric analysis has also previously been reported by other dynamic studies to decipher the drug-cell interactions dynamics mounting the previous evidence (49–51). This preliminary study is a pilot for further translational studies to recapitulate the BBB *in-vitro* and to test drug-BBB interactions and dynamics of BBB cells as a future prospect of our research team. This proposed BBB model could act as a future backbone for evaluating the efficacy of treatments for glioma and the passage of drug cargos through the BBB for testing cutting-edge glioma theranostics. Treatment of gliomas has long been a major challenge of neuro-oncology due to the tumor heterogeneity and the diffuse and infiltrative nature of the growth of the high-grade gliomas and hence much attempt has been devoted to improving the efficacy of glioma treatment comprising using external beam radiation or external beam radiation and soferanib (52, 53). This proposed BBB model could aid in improving translational research on the impact of external beam radiation or other chemotherapies for glioma treatment in the future. Moreover, using this blood-tumor barrier (BTB) model could aid the introduction of novel therapeutic biomarkers that predict optimized response to therapy. Prediction of therapeutic responses for oncologic purposes possesses great significance when translated into clinical stages for all types of cancers (54, 55). this model could serve as a future backbone for studies focusing on the interactions of the therapies on BTB and the biomarkers predicting tumoral cell death and therapy success in future oncological practice.

## Data availability statement

The original contributions presented in the study are included in the article/supplementary material. Further inquiries can be directed to the corresponding author.

## Ethics statement

The animal study was approved by NIMAD committee(IR.NIMAD.REC.1398.047) and also IUMS ethics committee by (IR.IUMS. AEC.1401.042). The study was conducted in accordance with the local legislation and institutional requirements.

## Author contributions

PS: Conceptualization, Writing – original draft, Writing – review & editing. MR: Conceptualization, Writing – original draft, Writing – review & editing. MN: Methodology, Writing – original draft, Writing – review & editing. FH: Conceptualization, Supervision, Writing – original draft, Writing – review & editing. A-RM: Conceptualization, Investigation, Writing – original draft, Writing – review & editing. DA: Conceptualization, Data curation, Formal analysis, Investigation, Methodology, Project administration, Resources, Software, Supervision, Validation, Visualization, Writing – original draft, Writing – review & editing.
